# Where Is the Client in Client-Centered Care? Centering the Experiences of Integrated Care Among Black Women in Public Health Settings

**DOI:** 10.3390/healthcare14030416

**Published:** 2026-02-06

**Authors:** Annalise J. Tolley, Victoria C. Scott, Jennifer Langhinrichsen-Rohling, Alicia A. Dahl, Ebony Rao, Jae Hoon Lim

**Affiliations:** 1 Department of Psychological Sciences, University of North Carolina at Charlotte, Charlotte, NC 28223, USA; vscott10@charlotte.edu (V.C.S.); jlanghin@charlotte.edu (J.L.-R.); 2 Department of Epidemiology and Community Health, University of North Carolina at Charlotte, Charlotte, NC 28223, USA; adahl3@charlotte.edu (A.A.D.); erao@charlotte.edu (E.R.); 3 Department of Educational Leadership, University of North Carolina at Charlotte, Charlotte, NC 28223, USA; jhlim@charlotte.edu

**Keywords:** delivery of health care, integrated, patient-centered care, public health, mental health services, phenomenology, multimethod design

## Abstract

**Highlights:**

**What are the main findings?**

- Quantitative satisfaction and cultural responsiveness metrics failed to capture nuanced integrated behavioral healthcare (IC) experiences and persistent unmet needs, which were revealed through qualitative inquiry.
- IC in public health clinics enabled critical access to mental healthcare for Black women with low-income and economically marginalized status (LIEM), but mental health needs remained unmet without follow-up and more hands-on support.

**What are the implications of the main findings?**

- Clients described person-centered (rather than client-centered) care as crucial for positive IC experiences, which requires providers and healthcare systems to holistically understand clients and to follow up with appropriate action.
- While IC facilitates essential mental healthcare access for Black women with LIEM status, its equity-promoting potential is undermined without closed-loop referrals, which may inadvertently compound institutional harm.

**Abstract:**

**Background/Objectives**: Integrated behavioral healthcare (IC) in public health settings may be optimal for advancing mental health equity among low-income and economically marginalized (LIEM) Black women. This study explores the provision of mental health services in public health clinics and assesses client experiences and recommendations for healthcare system improvement. **Methods**: Black women receiving mental health services in public health clinics completed surveys (*n* = 81) and in-depth interviews (*n* = 8, follow-up: *n* = 7). Analysis included descriptive statistics, interpretive phenomenological analysis, and member checking (*n* = 4). **Results**: Survey respondents reported high perceptions of providers’ cultural responsiveness (M = 13.35/14) and high satisfaction with IC services (M = 4.48/5.0). However, qualitative interviews revealed that these scores may be relative to low baseline expectations for care. Across interviewees, personalized care emerged as critical for high-quality IC service delivery, and pregnant interviewees reflected on the importance of IC during pregnancy, which can compound prior mental health concerns. Notably, positive IC reflections waned over time, and 75% of interviewees “fell through the cracks” between receiving referral for and accessing community resources, resulting in persistent unmet mental health needs. This experience, paired with a lack of systemic follow-up from the public health department, was perceived as a form of institutional betrayal. **Conclusions**: While IC in public health settings holds promise for health equity, results underscore the need for person-centered care that prioritizes authentic screening, warm handoffs, and closed-loop referrals—particularly for LIEM Black women, who frequently have prior experiences with fragmented healthcare systems. To ensure IC meets client needs without causing unintentional harm, healthcare systems should co-design solutions with clients.

## 1. Introduction

In the United States (U.S.), Black women with low-income and economically marginalized (LIEM) status are at a higher risk of depression than their white, affluent, male counterparts [1,2,3], and they also bear the highest disease burden [4]. This disparate burden is driven, in part, by underutilization of mental health services [4,5,6] due to a plethora of psychological and structural barriers amidst the interplay of classism and racism. For example, the underdiagnoses of depression among Black women as compared to their white counterparts [7,8], stigmas surrounding mental health disorders and help-seeking behaviors [9,10], geographic and financial inaccessibility of mental health services [11,12,13], and inaccessibility of high-quality care that is client-centered and culturally responsive [14,15]. Lack of transportation, resource-constrained health systems, lack of insurance coverage, and limited mental health providers—all more common in communities with greater populations of Black individuals [11,12]—make it especially difficult to access specialized services like high-quality behavioral health care.

Public health clinics are key locations to integrate behavioral health services and may be uniquely suited to meet the mental health needs of populations with limited access to services. Public health clinics offer cost-effective and accessible care to low-income and economically marginalized clients by serving those who are without insurance, are underinsured, have Medicaid, or have Medicare. This makes public health clinics potentially optimal settings to promote health equity by meeting the mental health needs of Black women with LIEM status through integrated behavioral healthcare (IC). IC refers to a collaboration between primary care and behavioral health clinicians who provide systematic, cost-effective, and client-centered care [16]. Three of integrated care’s defining features–holistic care and wellness, cultural responsiveness, and client-centered care–may be especially important to meet the mental health needs of Black women with LIEM status. Yet healthcare thought-leaders admonish that the promise of IC as revolutionary remains unfulfilled [17,18]. This is partially due to IC being detached from the client perspective in both research and practice.

While qualitative research methods are particularly well-suited for understanding the client experience due to the ability of these methods to generate ‘thick descriptions’, the majority of IC studies utilize quantitative methods [19]. In a scoping review, Youssef and colleagues [19] identified only 12 studies internationally that sought to understand IC experiences with mixed or qualitative methods. This warrants a prioritization of these methodologies within IC research. In addition, the IC experiences of Black individuals and/or those with LIEM status are underrepresented in the IC evidence base, regardless of methodology [19,20,21]. For example, in a systematic IC review of randomized controlled trials (RCT) between 2000–2015, only four studies included samples of 50% or more Black individuals. Of those, just two focused on the implications of IC specifically for advancing Black health. In Youssef and colleagues’ scoping review [19], only two U.S. studies sought to understand the integrated care experience of LIEM samples [22,23], and only one U.S. study recruited a majority Black sample [24]. While this study does not address the RCT gap, it meaningfully advances IC literature by centering the voices of Black women with LIEM status through mixed methods.

While there exist a number of hypothetical client-centered models for IC [25,26,27], the extent to which these are applied in practice remains to be seen within the literature. This knowledge gap is particularly important in the context of social responsibility within public health systems. If public health clinics are designed to promote community wellness, then these clinics have an ethical imperative to collect feedback directly from the supposed beneficiaries of care. Soliciting the client perspective and integrating it into healthcare labor practices is critical for a healthcare system’s ethos, accountability, and long-term sustainability [28]. This study, which culminates with recommendations for client-centered IC in practice, will contribute to these aims among healthcare practitioners.

## 2. Materials and Methods

### 2.1. Integrated Behavioral Healthcare Setting

This study took place as part of an integrated behavioral healthcare initiative within an urban public health department in North Carolina, U.S., known as the Holistic Opportunity Program for Everyone (H.O.P.E). H.O.P.E. was designed to integrate behavioral health services into Supplemental Nutrition Program for Women, Infant, and Children (WIC) and Adult Health clinics via three services: (1) depression symptom screening, (2) referral to and consultation with a Behavioral Health Provider (BHP), (3) and referral to mental health and community resources.

Clients were screened for depressive symptoms during their routine care visits using the Patient Health Questionnaire-2 (PHQ-2). In WIC clinics this was conducted by the nutritionist at a time within the nutritionist’s discretion. In Adult Health clinics the PHQ-2 was conducted by the primary provider at the beginning of the visit. Clients who scored positive on the PHQ-2, or who were identified using clinical judgment, were referred to the BHP (PHQ-2 cut-scores for Adult Health clinics and WIC clinics were three and two, respectively. While three is traditionally used [29], additional research on perinatal populations revealed that lowering the cut score to two increased the sensitivity of the screening tool to identify more women in need [30,31,32]. This cut-score change could not be implemented in Adult Health clinics due to limitations of the Electronic Health Records system). During the behavioral health consultation, the BHP conducted the PHQ-9 to more comprehensively assess depression symptoms. Consultative services also included active listening, helping clients apply to public services, and referring clients to mental health and community resources. These referrals were tailored to clients’ needs and included low-cost therapy clinics, affordable child support options, breastfeeding consultations for new mothers, etc. The majority of clients served by H.O.P.E. were Black women and Hispanic women, all of whom were LIEM in order to qualify for public health services (i.e., eligibility for WIC and Adult Health services requires that families have an income of less than 185% of the U.S. Poverty Income Guidelines). For additional details on H.O.P.E. implementation, please refer to [33].

### 2.2. Theoretical Orientation

This study was situated within a social constructivist paradigm, in which multiple realities exist and are constructed by integrating social, cultural, and historical contexts [34,35]. To understand client experiences within context, remain open to any themes that arose, and co-imagine solutions with clients, this study utilized phenomenology. Phenomenology seeks to identify the “essence” of a particular phenomenon. Because the essence is “knowable only through embodied perception” [34,36], phenomenology requires in-depth data collection from a distinct sample for whom the research question is relevant. It has a precedent of being utilized by health psychologists to understand complex healthcare experiences [37], which suited it for this study. Phenomenology identifies commonalities across experiences, but by prioritizing idiographic accounts in analysis, it honors in-group heterogeneity and allows for the voices of participants to be adequately presented in results. This mixed-methods study was qualitative dominant, but it included quantitative data to enhance generalizability.

### 2.3. Participants and Procedure

All clients were recruited from H.O.P.E. with the support of the county’s public health department. Survey recruitment was conducted in-clinic and by email with the support of public health providers and research team members. Eligibility for survey participation included the following: ≥18 years; female; client at one of the five public health clinics; received one or more H.O.P.E. services; can read, write, and speak in English or Spanish. Confidential surveys were completed online via Qualtrics or on paper to make the survey accessible to those without smart phones. Respondents could take the survey once and were compensated with a $10 Amazon gift card. The survey averaged 5–10 min to complete.

Interview participants were recruited through the survey or through BHP referral. For eligibility, participants needed to receive services from a BHP and identify as Black (the demographic of women primarily serviced by H.O.P.E.). A purposive sample of eight was recruited in accordance with rigorous Interpretive Phenomenological Analysis [38,39].

The first interviews (50–90 min) were conducted on Zoom using a semi-structured interview guide. Participants received a $50 Amazon gift card for their participation. Follow-up interviews (15–60 min) were conducted based on participant interest and availability for additional compensation, a $10 Amazon gift card.

The follow-up interview was unstructured. The only objectives were to understand if participants accessed community referrals and, if so, the degree to which those referrals met their needs. It took place 1–3 months after the initial interview. After preliminary qualitative analysis, interview participants were invited to participate in member checking [40] during a 20-min one-on-one Zoom session using an unstructured interview guide (Appendix C). Themes were presented to participants, and they were asked to reflect on whether the theme captured their experience. They were compensated with an additional $10 Amazon gift card. This process was intended to increase the trustworthiness of the research.

As long-standing collaborators with the health department, we abided by the department’s ‘Mental Health Crisis’ policy. If clients presented an imminent threat to themselves or to others, they would have been referred to a qualified professional for mental/behavioral health services (assessment, de-escalation, linkage to crisis service, follow-up care).

#### Data Management

Raw survey data were stored on an institutional Qualtrics account with two-factor authentication (2FA). Only the primary researcher had access. Upon download, any identifying information used for the gift cards was removed by the primary researcher before data were uploaded into an institutional Google Drive with 2FA for analysis.

Raw interview data were stored in a password-protected Otter.AI account (https://otter.ai, accessed on 2 August 2023). Transcripts were edited in Otter.AI for accuracy before being uploaded to the institutional Google Drive for analysis, which was made accessible only to research team members approved by the institutional review board.

### 2.4. Materials

#### 2.4.1. Survey Materials

The survey is available in Appendix A. Demographic survey items captured age, gender, race, ethnicity, services received, and location of services. To assess client satisfaction with H.O.P.E-specific services, clients were first asked to select which H.O.P.E services they had received (Appendix A, Question 1). After making their selection, they were asked: “Based on the services you circled above, how satisfied or dissatisfied are you with these specific services?” An additional satisfaction question was asked without prompting clients to anchor their response in H.O.P.E services: “How satisfied or dissatisfied were you with the overall services you received while at the clinic?” Answers ranged on a 5-point Likert-type scale from strongly dissatisfied to strongly satisfied. Cultural responsiveness was assessed with the Clients’ Perceptions of Providers’ Cultural Competency Instrument (CPOPCC) [41]. The scale includes three constructs–promotes supportive and meaningful interactions, promotes connection with others, acts on behalf of others—via 22 items (omnibus α = 0.89; [41]). Partial scales were used to reduce response burden. Fourteen items were selected by the research team by considering the items with the highest eigenvalues from Pacquiao and colleagues’ validation study [41], the relevance to the research questions, and the relevance to the integrated care context. Responses were on a Yes/No scale.

#### 2.4.2. Interview Materials

The interview guide was developed through close collaboration with public health administrative leadership and the BHPs. It included nine core questions and additional probes (Appendix B). The following experiential domains were assessed: (1) depression screening process; (2) referral process; (3) consultation with the BHP; (4) accessing resources provided by the BHP.

### 2.5. Analysis

#### 2.5.1. Survey Analysis

Survey data were downloaded from Qualtrics and descriptive statistics were calculated in Excel. Sample characteristics were calculated with frequencies. For Likert-type questions, the mean and standard deviation were calculated. Yes/No scales were coded as 1/0. Answers were summed, and the average and standard deviation were calculated.

#### 2.5.2. Interview Analysis

Interpretive Phenomenological Analysis (IPA) was employed for this study [42]. IPA elucidates the systemic and common properties of human experiences in a specific sociocultural context while preserving individual uniqueness. Audio files were transcribed verbatim using Otter.ai and audited for quality. Any identifiable information was redacted from the interview transcripts, and each participant selected a pseudonym for reporting. The primary investigator (A.J.T) used IPA’s seven-step approach [42] for data analysis, which was completed manually on Google Docs. Two cases were piloted with an IPA subject matter expert (J.H.L) to ensure quality. Feedback was integrated, and all eight cases were reanalyzed, converging into five emerging group experiential themes (GETs). Before finalizing the GETs, member checks were completed. The researcher asked for feedback on each emerging theme to determine if it adequately captured the participant’s experiences. The member checks also revealed and clarified outstanding gaps in the researcher’s knowledge. This process informally shaped the GETs and provided additional nuance to the findings.

## 3. Results

### 3.1. Participant Characteristics

#### 3.1.1. Survey Participants

The survey was distributed between May 2023 through January 2024. One hundred and thirty-four women began the survey, with 128 finishing it (96% completion). For the purpose of this study, a subsample of respondents—those who identified as Black (*N* = 81)—were included in analysis. All respondents completed the survey in English. Of the five clinics, two provided the majority of respondents: 31 (38%) and 32 (40%). The other sites provided 2–7 respondents (2–9%). Seven respondents received services from multiple sites. Additional characteristics are reported in Table 1.

#### 3.1.2. Interview Participants

Eight Black women between the ages of 19–39 participated in the interviews (Table 2). Half of the women received WIC services and half received Adult Health services. Seven women participated in the follow-up interview, and four women participated in member checks.

### 3.2. Client Satisfaction

In aggregate, respondents reported high satisfaction with public health services overall (M = 4.58, SD = 0.65) and with H.O.P.E.-specific services (M = 4.48, SD = 0.86) on a five-point, Likert-type scale. A subsample was constructed of clients who reported a consultation with the BHP (*n* = 20). These women also reported high satisfaction with both public health services overall (M = 4.30, SD = 0.98) and with H.O.P.E.-specific services (M = 4.30, SD = 1.03). A subsample of the interview participants (*n* = 8) was constructed to compare satisfaction scores with the overall sample. Satisfaction results were similar for both public health services overall (M = 4.38, SD = 0.74) and with H.O.P.E.-specific services (M = 4.38, SD = 0.74). More insights related to client satisfaction are reflected in the Group Experiential Themes.

### 3.3. Perception of Cultural Responsiveness

On average, clients reported that services were culturally responsive (Table 3). Of the 14 cultural responsiveness items, clients selected “yes” an average of 13 times (M = 13.35, SD = 1.06), indicating that respondents felt positively about their care. This held true across each subscale. Within the subsample of clients who reported a consultation with the BHP (*n* = 20), high levels of cultural responsivness were also reported (M = 12.65, SD = 1.39) though slightly lower. Within the subsample of the interview participants (*n* = 8), high levels of cultural responsiveness were also reported (M = 11.75, SD = 1.67). However, interview clients, on average, endorsed one item fewer than the overall group.

To integrate quantitative cultural responsiveness results with qualitative insights, three of four member-check participants were asked about cultural responsiveness in healthcare settings. Jade was asked what culturally-responsive care looks like to her. She began her answer by describing what it does not look like: demographic checkboxes. She believed that society’s emphasis on demographic characteristics may increase discomfort among historically marginalized groups due to fear of “being penalized”. In her view, cultural responsiveness meant that social identities are secondary to being seen as a whole person:

*If we send it back to, ‘we’re humans and we all are affected,’ then I also feel like people will feel a little more comfortable. Like, ‘okay, they don’t even want to know what race I am. They don’t even want to know my gender. They don’t even want to know my sexual orientation. They just want to make sure I’m okay.’ And that’s most of the time what people want. Just make sure I’m okay. Like, help me live, live my life to the fullest. That’s what I want. Don’t take that away from me.*

Diamond concurred: “whether they are flamboyantly gay, or, I don’t know, be Black and ugly, who cares? It doesn’t matter. Just see this person as a person that is looking to you for help”.

Daphanie was asked whether culturally-responsive providers were important to a positive IC experience, to which she replied affirmatively. She wanted her provider “to understand [her] background”. Within the conversation surrounding culturally-responsive care, both Daphanie and Diamond brought up racially-incongruent providers (unsolicited). Daphanie described historically wanting Black therapists, who she felt had better understood her in the past. However, after a positive experience with a white therapist, she realized that there were other ways she could connect with her providers: “even though we weren’t the same color, she understood me and where I was coming from, as in like, being a bisexual woman”. She went on to describe other differences between her and her therapist that were overcome because of her therapist’s curiosity and self-disclosure: “she gave me not just my life; she gave me some of hers, too”. In her final reflections, she remarked that cultural responsiveness was about providers seeking to understand their clients lived experiences, but also about reciprocity:

*[When] they’re trying to be relatable to you, it gives you that feeling of actually trying. To be like ‘okay you’re actually trying to understand me’. So it gives you that cultural competence. Because it’s like, you’re trying to take my culture and also telling me about yours, too.*

When she felt that curiosity and reciprocity, she felt “completeness as a person”.

### 3.4. Client Experience

Six of eight (75%) women reported a positive screening experience, while one indicated a neutral experience, and another indicated one positive and one negative experience. Most of the women (*n* = 5, 62.5%) reported positive referral experiences, while one woman reported a negative experience, one reported a neutral experience, and one reported a mix of positive and negative referral experiences. Resources provided to the clients varied, though all women were provided a therapy referral to mental health services such as Mental Health of America or the Hope Community Clinic. Three women had accessed community resources at the time of follow-up, with only two women accessing mental health resources. Participants reported a myriad of perceived barriers to mental health care, with transportation and insurance/cost being leading barriers. The group experiential themes (GETs, [31]) are summarized in Table 4.

#### 3.4.1. Theme 1: On My Terms

Depression screeners were increasingly seen as a routine part of care, but clients responded on their own terms. For many, like Justinia (20, a new mother-to-be) and Jade (30), the depression screener was a normalized aspect of healthcare encounters. Justinia stated: “it wasn’t my first time being asked [screening questions]. I think every provider that I’ve seen…they all tend to typically ask that. I kinda like it”. Conversely, clients like Diamond (32, a regular client at the public health department) and Noelle (25, a client new to town) expressed surprise. While Diamond felt that the depression screener was a pleasant surprise and put her at ease, Noelle was “shocked”. A depression screener during a primary care visit was inconsistent with her and her friends’ past healthcare experiences and was thus unexpected. As a private person who struggles to trust others, Noelle felt that unexpected services infringed on her closely guarded privacy:

*It felt a little weird, because I really didn’t know anybody there. And I didn’t expect them to ask me about my emotions and things like that… [Other people] just pretty much just gave me the impression that you go in, you sign your papers, they talk about the WIC benefits and how it works, and if you’re approved or not… Nobody mentioned a [supplemental iron] shot or being weighed or anything about counseling or therapy.*

Regardless of their expectations for mental health screeners, all women described that answering honestly came down to personal agency. Both Jade and Nina (33, full-time employee and mother of six) disclosed their mental health concerns even prior to receiving the screener, indicative of their own drive to get help. Jade indicated, “I already prepared myself: whoever it was, I was going to ask [for help] … I went with the purpose”. In contrast, Sandra (late 30s, background in healthcare) had no intention of disclosing. She made the decision to do so only because of her provider’s “welcoming spirit”. For clients who need mental health support but may be weary of disclosure, she noted the importance of using screeners “at the end [because] a person can say whether or not they want to answer these questions *with this person*”.

#### 3.4.2. Theme 2: Better than Bad

While five of eight women explicitly said that their IC experience at the public health department exceeded their expectations, this satisfaction was relative to having no or low expectations for care. For Jade, who felt that no one wanted to go to the “gloomy” health department, her experience with H.O.P.E. made her feel like “the good outweighs the bad… it’s not such a bad experience, when you’re able to get what you need”. Diamond, who had a negative experience with the public health department in the past, said “it’s definitely different from the last time I had been, and it felt a little bit better”. Noelle, who formed her care expectations around out-of-state care, was pleasantly surprised that the local public health department provides “resources that someone actually needs”, which is “very helpful compared to where I come from”.

Diamond, Jade, and Justinia indicated that their low expectations were due to their negative perceptions of the public health department. Diamond associated the predominantly Black and Hispanic clientele with low quality, fearing “they about to mix my results up with somebody else’s crap”. The clientele, the location (“It’s a little hood”), and the free services (“I wish I could go see [another doctor] and afford to get his opinion”) all signaled low-quality care to Diamond. While less explicit, Jade and Justinia shared similar biases. Jade said that she and her friends avoided the public health department: “Nobody wants to go to the health department, but you got to do what you got to do”.

In addition to public health department stigmas influencing expectations, some women demonstrated a general bias against government-funded agencies. This is a special consideration for IC in the public sector:

*I’m a person that realized certain expectations of certain things are just null and void…Do I have expectations for my children? Yes. Expectations for these programs to be all that I need? No… I don’t have expectations, especially when I’m dealing with government entities.*

Nina also expressed her distrust towards government entities.

*[The] county drops the ball a lot. Because it seems like, if they had the option, they really don’t care [about people], which is fine. You don’t have to. But then what do you do with all the money that you actually get from the government?*

This bias against government agencies emerged consciously and perhaps also unconsciously (i.e., two women compared negative healthcare experiences to the DMV).

In other instances, low expectations for IC at the public health department were driven by past experiences. Daphanie (27, been unable to access counseling services for two years) describes her first experience with the depression screener at the public health department as feeling perfunctory. She also reported never receiving a callback from the BHP despite screening positive for depression symptoms. This experience, paired with another privately-owned healthcare system overpromising but underdelivering on mental health services, shaped her expectations for care. She felt that she was not being taken seriously and had to prove that she needed help.

*They’re not understanding that you’re really trying to get this help. And they just over here, like rescheduling your appointment every time they get the appointment. No, bruh. Like this is the third time…Honestly, I’ve been here for almost two years. I’ve been trying… [my provider] was like, ‘so do you want them to reach out to you? Do you want to reach out to them?’ I think we can do both ways. Just so they know I’m serious. I’m serious about it.*

#### 3.4.3. Theme 3: Falling Through the Care Cracks

H.O.P.E. offered three primary services: (1) depression screening, (2) referral to and consultation with a BHP, (3) and referrals to mental health and community resources. The fourth care domain (though outside public health department purview) was whether or not clients accessed the resources. Two subthemes emerged to describe where clients fell through the cracks. First, between the depression screener and the referral to/consultation with the BHP. Both women who experienced this crack highlighted the consequences of this error. After screening positive for a number of depression symptoms, Diamond waited 30 days to receive a callback. She described the delay as “shameful” and foreboded that the consequences could have been fatal:

*They definitely should have put me with someone else immediately… I would have been, done killed myself, had the funeral, and at Heaven’s gates… I don’t think I have 30 days to really just get a grip on my mental [health].*

Daphanie also experienced this care crack, though she did not receive a callback at all. It was not until months later, when she returned to the public health department, screened positive again, and accepted another referral, that she finally heard from the BHP. When asked why she decided to still answer the screener honestly, Daphanie’s response illustrated an inner tension between a need for IC (as a point of entry to mental health care) but an eroding faith in its ability to get her services.

*I’m not fine. I’m tired of that—I’m tired of lying to y’all. Nope. I’m bout going crazy over here. So, it’s like, nah, I would never lie when they ask that question. But I do pull back on seeking help. Because it’s like you keep trying. And it’s not going nowhere.*

These testimonies contextualize why half of the interviewees (Diamond, Daphanie, Jade, and Sandra) explicitly called for more on-site support as a mechanism of improvement both for their own experience and for the experience of other women. Daphanie also emphasized that on-site behavioral health services may be timelier, which is critical for women suffering from depression: “This person is asking for help, but they might, by the time you talk to them again, they might change their mind”.

The second crack (and the second subtheme) emerged after receiving referrals, when clients were no longer considered within the public health department’s purview. Only three of eight women made it to community resources, with only two of those women accessing therapeutic services despite a high need among all women. The follow-up interviews elucidated that many women were unable to benefit from their referrals due to nebulous communication within the triangle of the client, the BHP, and the community resource referral agencies. For example, both Daphanie and Diamond were told by the BHP that they were scheduled with Mental Health America, but they did not know for when nor did they ever receive a callback from the referral agency (unbeknownst to the BHP). Diamond describes this lapse: “I slipped through the cracks”.

Nina saw the “miscommunication” between a community resource referral agency and the BHP first-hand. She was on the phone with the BHP while she called a local food and diaper bank on Nina’s behalf. The diaper bank said that they had diapers, but when Nina went to pick them up, there were none available. As for her therapeutic resources, Nina wanted a therapist who would take her insurance. She was waiting to hear back from the BHP, who said she would do additional research to find someone in-network: “I said I’ll give her a week to find a resource that takes my insurance. Because by the time the week is up, you know, I’ve moved on… it’s no longer in my power”. At the time of the follow-up (a few months later) she had not heard back.

There were various opinions as to how much onus the client should carry in order to get support. As evidenced by Nina’s previous quote, she felt like she did her part by asking for help. The remaining responsibility was in the hands of the healthcare system. In contrast, Noelle saw herself as needing to do more:

*I’m not gonna hold [the BHP] accountable for anything…It’s up to me to pretty much reach out to the resources and take the help that I need. But I haven’t really gotten a chance to do it; because I’m holding back, or something is stopping me, or whatever the case may be. So, I feel like she was a good connection, but it’s just me, that’s the issue.*

Noelle’s testimony felt pained and seemed to be impacted by her depression symptoms. She had only minimally left the house in a year, had no local family or friends, and had recently given birth to her first child at the time of follow-up. She blamed herself for never getting connected to resources. Jade, another client and former social worker, warned of this outcome in her interview. She advocated for more hands-on support when clients are depressed and in crisis. She warned that a clients’ depression symptoms (e.g., lack of motivation, fatigue) create “room for error” when tasked themselves with referral agency outreach. Daphanie felt similarly and evoked a need for paternalism on the part of the healthcare system: “If this person has already told you [they need help] just do it for them; don’t give them the option”. Sandra agreed that on-site support was valuable for busy moms, regardless of mental health crisis; “let’s be real. It’s all about convenience”.

#### 3.4.4. Theme 4: Not Another Number

Clients described high-quality IC as grounded in a provider’s ability to demonstrate authentic compassion. This was perceived by clients in three ways, which informed the following subthemes: nonverbal cues, tailored services, and follow-up. As it relates to nonverbal cues, Justinia emphasized the importance of eye contact during mental health screenings: “It just shows me that you actually care, and you’re just not following routine and protocol”. Daphanie also alluded to the importance of nonverbal cues. While she did not identify specific behaviors, she contrasted two of her depression screening experiences to highlight the different ways providers can make clients feel while performing the same service. Her first screening felt quotidian and dismissive, as if the provider “just wanted to get me in and get me out”. But in her second experience she described,

*You could tell that [my provider] actually cared, even though we just met that day…I ended up crying…You just sense the genuineness, and so that made it easier to be able to actually speak up.*

Tailored services during the BHP consultation was also reported as key to clients’ perception of high-quality services; it was organically brought up by seven of eight women. Sandra described tailored referrals as a signal that she was heard: “everything that I told her I needed, she sent me a referral for…It wasn’t off…which means she understood what my requests were”. This was evident from clients’ perceptions and from their actual referrals. For example, Mabel (19) was the only woman referred to On Ramp Resource Center, a hub of resources for 16 to 24-year-olds which includes therapy, social workers, spiritual guides, GED support, and financial literacy training. Justinia, a young pregnant mother, was connected with a community baby shower and a free program that pairs registered nurses with women throughout their pregnancy. Noelle, who did not have a valid license and relied on public transportation said her BHP “went above and beyond” by choosing resources that were geographically accessible.

Culturally-responsive care was not explicitly named. However, clients did seem to reference the need for both culturally-responsive providers—who can “understand you for your background, and your understanding, and culture, and everything”—and culturally-responsive services—“you have to deal with women from different cultures… for a lot, [asking mental health questions in WIC waiting rooms] would not make sense for those who have husbands who are very involved”.

Finally, clients indicated that follow-up was critical for authentic, high-quality care. Among the six women who did not access resources post referrals, all six believed follow-up would have improved their experience. Without closed-loop services, “you left me with the tools, but I don’t know how to use them.” Improving resource utilization among clients is important for healthcare system impact, but clients reported it is also important for organizational reputation. When asked what she would change about her IC experience, Nina immediately said, “check in more” to elicit the positive feeling of a “hey, just thinking of you” text message from a friend. Nina’s reference to a friend demonstrates how healthcare interactions can feel personal. This phenomenon came up during Daphanie, Diamond, and Sandra’s interviews—all of whom did not access resources. When Daphanie was asked why follow-up was important to her, she compared her healthcare experiences to a pattern of relationships without reciprocity:

*I done learn that I like people to care about me consistently…I tend to give people my all the whole way out. And that’s what ends up hurting me the most. Because I’m giving you my all, and I’m really not getting nothing back.*

Jade echoed this emotional sentiment during the member check. To her, no follow-up felt like a “slap in the face.” Sandra felt similarly and questioned whether behavioral health services without follow-up were really for clients or just for government “kickbacks”. In summary, clients reported that authentic, humanizing care was foundational to high-quality care. This was achieved through nonverbal cues, personalized services, and follow-up. In Daphanie’s words, those qualities “made me feel like I wasn’t just another number. And that’s something for me… I want to feel like a human being”.

#### 3.4.5. Theme 5: Overlooked and Adding up

Four women in the sample received WIC services and were pregnant during their interview. All discussed how their identity as a mother-to-be interacted with their preexisting mental health concerns. Justinia and Noelle had both been struggling with depression for many years, but this was the first time that they struggled with depression while pregnant. For both women, their pregnancy disrupted their previous strategies for wellness promotion. Justinia described stopping her depression medication “cold turkey”, out of fear that it would harm the baby. In the absence of medication, she searched for natural supports to promote her wellness, such as walks and attending cookouts at her Grandma’s church. She reflected, “I have to give myself even more grace because I’m not only dealing with what I was dealing with before, but also added hormones”. Noelle similarly struggled to promote her wellness in ways that had worked well in the past. She is a creative woman and loves painting shoes. However, the fumes from the paint were not safe for the baby, so she lost an important natural support.

Justinia and Sandra both discussed how societal pressures to celebrate pregnancy can compound the penchant to suffer in silence. Sandra was recently widowed, and she described the grief that she felt undergoing her pregnancy for the first time alone: “not everybody is joyous about a pregnancy; it doesn’t mean that they don’t want the baby; doesn’t mean that they don’t love the baby. There’s just certain fears…”. She went on to explain how IC within WIC clinics can be especially important to fill a nonjudgmental support gap:

*[Pregnant women] may not have people that they can express certain feelings to safely… So now if you know [a provider] is there…they get a little taste of how they can have an outlet for what they’re feeling during this pregnancy.*

Justinia concurred, “I feel like pregnancy can sometimes be a little glorified. Like, ‘oh, your skin’s gonna glow; you’re gonna glow; your nails are gonna grow—look healthy.’ Um yeah, like I’ve seen the downsides of pregnancy”. She expressed her appreciation for conversations around depression and pregnancy: “It’s really important to highlight mental health during pregnancy because you know, people just emphasize postpartum depression and mental health a lot… it’s not just about postpartum”.

## 4. Discussion

**Figure 1:**
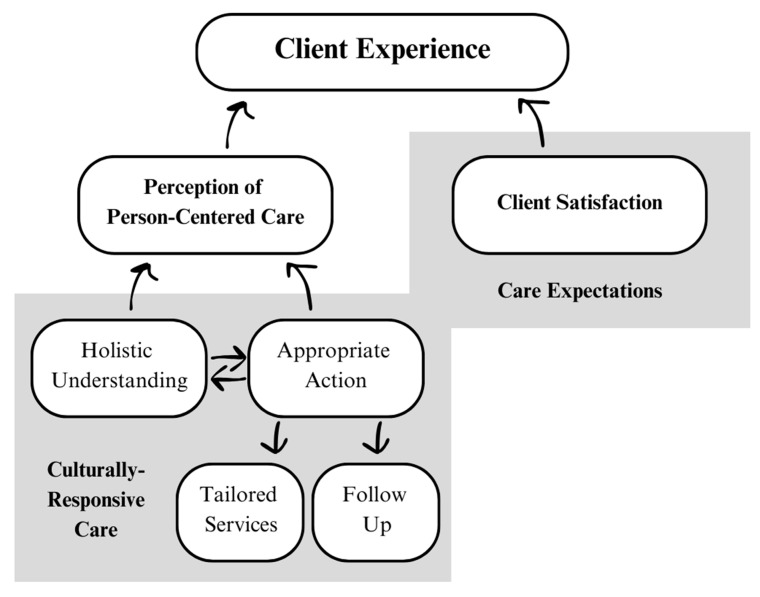
Emergent Elements of the IC Client Experience. This figure illustrates two factors that emerged as part of the client experience in this study. The dark gray boxes represent the context in which the other concepts were situated. For example, client satisfaction was situated within care expectations.

This study explored how Black women with LIEM status experienced behavioral health services in public health settings. There were two components that emerged as central to clients’ sensemaking of their IC experiences: person-centered care and client satisfaction (Figure 1).

### 4.1. Person-Centered Care

Person-centered care was central to a positive client experience. Person-centered care “focuses on the individual within multiple complicated contexts, including family and/or important others…[it] is holistic, individualized, just, respectful, compassionate, coordinated, evidence-based, and developmentally appropriate” [43]. It can be evaluated through questions such as “does your PCP know you very well as a person, rather than as someone with a medical problem?” [44]. Though person-centered care is related to client-centered care, scholars contend that it is more holistic and inclusive than client-centered care [44,45,46]. Person-centered care seeks to understand the person, the ecology in which they are embedded, and how the diagnosis interacts with life course experiences. Therefore, it is more likely to include family and community systems [45,46].

Across both screening and consultation, two primary factors that shaped clients’ perception of person-centeredness were (a) the process used by providers to understand the whole person, and (b) the actions taken based on that understanding. Regarding process, clients reported the importance of eye contact, being asked the screener questions in an empathic rather than perfunctory way, a holistic mental health assessment, and acknowledging client uniqueness. In order for providers to build a holistic understanding of their clients, women participating in member checks touched on the importance of culturally-responsive care, particularly when their provider was racially incongruent. They described culturally-responsive care as humble and reciprocal. Even though providers may not understand clients’ lived experiences first-hand, if they exercised curiosity and were willing to share pieces of themselves with clients, these differences could be overcome. This approach to holistic understanding also informed whether providers followed with appropriate action. Appropriate action looks differently based on personal preferences and cultural norms, which is why understanding interacts with action (Figure 1). The need to elevate cultural responsiveness in person-centered IC for racially and ethnically marginalized populations aligns with the CRASH model [47].

Consistent with another qualitatively-derived integrated framework on IC experience [26], providing tailored services was the action most frequently reported as signaling person-centered care. For example, one client in the study reported an appreciation for bereavement services provided for herself *and* her children. These actions go beyond active listening and respectful engagement. Tailored services demonstrate that providers will adapt to their client’s needs, rather than the client needing to adapt to the healthcare system. This person-centered approach subverts traditional provider-client power hierarchies, making it critical for empowerment and health promotion among historically marginalized individuals.

No follow-up (a form of inaction) was most frequently reported as undermining a perception of person-centered care. The majority of women in need of mental health services did not get connected to them in this study. Without additional support from the public health department, their previously positive experiences with IC were soured. This finding is especially important within the context of culturally-responsive care. Racially and ethnically marginalized women frequently report a history of being let down by their care [15,48,49]. Therefore, being given potential services without institutional follow-through may unintentionally compound feelings of institutional betrayal (i.e., institutional actions which either fail to prevent harm or perpetuate harm upon those reliant on it to meet their needs [50]). This was supported by this study’s results, which illustrated how clients felt personally betrayed by no follow-up despite their demonstrated need.

### 4.2. Client Satisfaction

In addition to person-centered care, client satisfaction is a salient—but noncomprehensive—component of the client experience. Despite very high satisfaction scores, clients described a number of ways in which their care was not meeting their needs. Within the “Better than bad” theme, clients described low to no baseline expectations for the public health department due to negative prior experiences and stigmas surrounding government welfare services. Therefore, even when only minimal standards of care were observed (e.g., test results not being mixed up with someone else’s, not waiting half the day to be seen), clients reported being highly satisfied. We expect that this finding would generalize to the larger sample, which reflected interviewee demographics and reported nearly identical satisfaction scores. By centering the experiences of these women, we saw that satisfaction was a relative concept [51] that may not indicate the presence of high-quality healthcare experiences [52,53], but rather the absence of overt harm. From a health equity perspective, high satisfaction driven by minimal expectations likely reflects internalized systemic inequities rather than programmatic success. To truly promote health equity, healthcare systems should invest in holistic evaluations of client experience that ensure a standard of care that can close gaps in mental healthcare quality between Black women with LIEM status as compared to their white, male, affluent counterparts [11,12,13,14,15].

Additionally, it is likely that satisfaction was time-bound. Due to the in-clinic recruitment strategy, clients reported satisfaction directly after receiving services. While clients may have reported immediate satisfaction based on their acute experience of feeling understood, interviewees described wavering satisfaction when not followed by long-term, appropriate action (Figure 1). If the IC program fails to bridge the gap between the first clinical encounter and providing tailored necessary services to meet women’s needs, the initial satisfaction score is likely overinflated. IC programs that seek to promote health equity should therefore invest in longitudinal assessment as an ethical imperative. Otherwise, acute satisfaction may overshadow the continued mental health burden experienced by Black women [4]. This is particularly important for Black women of LIEM status, who often have fewer options for affordable, accessible, and culturally-competent care [11,12,13,14,15].

### 4.3. Future Directions

#### 4.3.1. Implications for Practice

Public health clinics may be key settings for IC, particularly with the aim of promoting behavioral health equity among Black women with LIEM status [54,55]. To improve IC implementation using client insights, the following recommendations were illuminated for practice.

1. **Leverage person-centered care to overcome mistrust;** Clients were skeptical of their providers and the public health department’s care quality due to prior experiences and negative perceptions of government systems; this poses a distinct challenge for IC programs that rely on mental health symptom disclosure within publicly funded, short-term care facilities. However, clients overcame their skepticism when they received person-centered care, which humanized the clinical encounter and overcame feelings of being treated like “another number”. To build rapid rapport, practitioners should prioritize empathic nonverbal cues (e.g., eye contact, offering tissues if someone is emotional), authenticity, and judicious self-disclosure. By moving beyond “demographic checkboxes” to co-designing tailored care plans, providers can overcome mistrust and advance health and wellbeing through culturally-responsive care.
2. **Prioritize IC throughout the perinatal period;** While mental health services for women often center on Postpartum Depression, clients suggested it can overshadow unmet mental health needs during pregnancy. Practitioners should be aware that pregnancy may disrupt disease management through the loss of medication and natural supports (e.g., movement, hobbies, social groups), and pregnant women may need additional support establishing a tailored treatment plan during pregnancy. Practitioners must also recognize that societal pressures to “glorify” pregnancy can lead Black and LIEM women to suffer in silence. To address this, IC systems must provide safe, nonjudgmental outlets for clients to process complex emotions and “downsides” of pregnancy without fear of being socially ostracized.
3. **Maximize IC impact through co-location and shared infrastructure;** According to SAMHSA [56], integrated care exists across six levels, ranging from minimal collaboration to full integration. While the H.O.P.E. program utilized co-located staff, siloed electronic health record systems—particularly between WIC and behavioral health—resulted in a “Level 2” functional status: basic collaboration at a distance [56]. This structural fragmentation created significant “care cracks” in which clients were easily lost during service transitions. To ensure institutional accountability, practitioners must move toward higher degrees of integration via coordinated information systems and on-site mental healthcare. In instances when external referrals are required, intensive hands-on support is critical, particularly when women are suffering from depressive symptoms and even seemingly small barriers may prevent service utilization. Higher degrees of integration are especially important for advancing health equity among Black women serviced by public health settings because, unlike their more affluent, white counterparts, they often have limited alternative routes to high-quality mental healthcare.
4. **Implement systematic follow-up to improve IC experiences over time;** Women reported feeling that the public health department was their primary entry point for mental health care, and without appropriate action on the part of the institution, they were likely to continue suffering in silence. Despite positive initial experiences with IC services, the majority of women interviewed were unable to access the resources they needed. With persistent unmet needs, a lack of follow-up from the public health department felt like a personal affront. We hypothesize that this finding can be explained by institutional betrayal (i.e., institutions not providing the preventive or supportive services for which individuals rely on them [50]). Instances of institutional betrayal do not occur in a vacuum and contribute to healthcare avoidance and underutilization of services [57,58,59] among the women most in need of equity-promoting services. IC programs that prioritize longitudinal continuity of care can combat institutional betrayal by demonstrating an authentic investment in client outcomes, fostering trust in public healthcare systems, and potentially repairing institutional harm—all of which are necessary for reducing health inequities faced by Black women with LIEM status.

#### 4.3.2. Implications for Research

This study adds to a growing body of research that indicates that client satisfaction is a myopic measure of client experience [52,53,60]. Yet, given the cost-effective nature of brief satisfaction surveys, these are still heavily utilized in healthcare settings. There are a number of challenges with implementing patient-reported outcome measures and patient-reported experience measures which have frustrated their widespread use [60,61,62], yet true understanding of the client experience is salient for service improvement and goals of health equity. Thus, researchers are tasked with (a) determining the domains that contribute to the client experience and (b) developing short surveys with content validity across each of these domains (e.g., [63]). Identifying a measure with conceptual complexity, administrative feasibility, and psychometric validity is important for future research.

Integrating behavioral health services into public health settings is an important systems-based solution to expand mental healthcare access and promote health equity. While gaining traction, IC in public health clinics is still understudied. Therefore, this study, which highlighted unique implementation considerations germane to public health settings, appears to be one of the first of its kind. To increase the widespread adoption of this promising approach, additional research should qualify the generalizability of our findings across different populations and degrees of care integration.

### 4.4. Limitations

There are some considerations to hold while interpreting the findings. First, due to ongoing construction at two of the clinic sites and a closure of one of the WIC sites halfway through implementation, there was an overrepresentation of survey respondents from two of the five public health clinics implementing H.O.P.E. Therefore, high satisfaction scores and CPOPCC may not be generalizable to experiences at other H.O.P.E. clinics. This also resulted in interviewees being recruited from the two overrepresented sites. Therefore, consultation experiences reflected interactions with one particular BHP. Their experiences may have been different with another provider. Second, many of the interviewees were recruited through the BHP. The BHP was asked to refer anyone who met the eligibility criteria of the study. However, it is possible that the BHP’s selection bias influenced which clients were referred. For example, the BHP may have selected clients (albeit unintentionally) because they were gregarious or receptive to services. This could have created systematic similarities among interviewees as compared to other clients who received consultations. That said, satisfaction scores and CPOPCC scores were not descriptively disparate between the interviewees and the other women who received consultations in the sample. Finally, the high perception of providers’ cultural responsiveness may reflect a limitation of the CPOPCC instrument and a potential ceiling effect. This is likely due to the dichotomous Yes/No response scale. Notably, the scale developers have since moved to a 4-point Likert-type scale with a “not applicable” option to increase variability [64]. Despite these limitations, this study provides valuable contributions for IC, health equity, and public health scholars.

## 5. Conclusions

This study began with a rhetorical question: where is the client in client-centered care? By centering the lived experiences of Black women with LIEM status, this research revealed that advancing health equity requires a shift from perfunctory client-centered models to holistic, *person*-centered care. While IC in public health settings holds immense potential for reducing mental health inequities, our findings suggest that without closed-loop referrals, equity-oriented IC programs may unintentionally compound institutional harm against Black and LIEM women who rely on these systems as their primary entry point for care. Furthermore, this study demonstrated that high satisfaction scores can be deceptive; for Black women with LIEM status, these metrics may reflect internalized systemic inequities and low baseline expectations rather than true programmatic success. To truly assess IC experiences, healthcare systems must move beyond acute client satisfaction surveys to more robust, longitudinal assessments of the client experience. While phenomenological inquiry is more resource intensive than the traditional methods of assessment in healthcare settings, the return on investment cannot be overstated. When IC research and practice invests in understanding clients, leveraging their strengths, and learning from their power, IC’s potential to promote health equity may be realized.

## Figures and Tables

**Table 1:** Characteristics of Survey Participants (*N* = 81).

Characteristics	*n* (%) ^1^
Age	
18–24	19 (23%)
25–34	44 (54%)
35–44	10 (12%)
45–54	4 (5%)
55+	4 (5%)
Total	81 (100%)
Ethnicity	
Hispanic	7 (9%)
Non-Hispanic	74 (91%)
Total	81 (100%)
Services Received	
Screener Only	57 (70%)
Screener & Referral Only	4 (5%)
Screener, Referral, Consult	20 (25%)
Total	81 (100%)

^1^ Percentages are rounded to the nearest whole number.

**Table 2:** Characteristics of Interview Participants (*N* = 8).

Pseudonym	Age	Services Received	F/U ^1^ Interview	Member Check
Jade	30	Adult Health	x	x
Diamond	32	Adult Health	x	x
Justinia	20	WIC		
Noelle	25	WIC	x	
Nina	33	WIC	x	
Sandra	Late 30s	WIC	x	
Daphanie	27	Adult Health	x	x
Mabel	19	Adult Health	x	x

^1^ F/U = follow-up interview. “x” indicates participation.

**Table 3:** Cultural Responsiveness Averages by Scale and by Sample.

Scale	Mean Sum ^1^ (SD)			Total Sum Possible ^2^
	Overall (*N* = 81)	Consult (*n* = 20)	Interviewees (*n* = 8)	
Promotes Supportive and Meaningful Interactions	7.90 (0.30)	7.70 (0.47)	7.63 (0.52)	8
Promotes Connections with Others	3.75 (0.58)	3.45 (0.89)	2.88 (1.13)	4
Acts on Behalf of Others	1.67 (0.55)	1.50 (0.51)	1.25 (0.46)	2
Total CPOPCC Score	13.32 (1.12)	12.65 (1.39)	11.75 (1.67)	14

^1^ Higher mean sum reflects the average number of times a client answered affirmatively. A greater mean indicates greater levels of perceived provider’s cultural responsiveness. ^2^ The total possible score is representative of the score if participants answered affirmatively to every question. SD = standard deviation.

**Table 4:** Summary of Group Experiential Themes.

Theme	Description
On my terms	5/8 clients were accustomed to depression screeners in primary care systems, but that did not mean that they would answer honestly. 8/8 women indicated that disclosing their mental health concerns was a matter of personal agency. Some women reported that they disclosed because they were overtaken by emotion while other women used a cost–benefit analysis.
Better than bad	5/8 women organically expressed that H.O.P.E. services had exceeded their expectations. However, 8/8 women reported having no or low expectations for their care. Reasons varied but included stigmas against the public health department, distrust of government agencies, and negative prior experiences with healthcare and welfare systems.
Falling through the care cracks Subtheme: Between screener and consult Subtheme: Between referral to and community resource access	H.O.P.E. offered three services: (1) screener, (2) referral to and consult with BHP, (3) referral to community and mental health resources. The fourth care domain was whether or not clients accessed their resource referrals, though tracking this was not within the public health department’s purview. Results indicated that 2/8 women fell through the cracks between the screener and referral/consult with BHP. In addition, 6/8 women fell through the cracks between referral and resource access. Overall, clients indicated needing more hands-on support.
Not another number Subtheme: Nonverbal cues Subtheme: Tailored services Subtheme: Follow-up	High-quality IC requires the provider to demonstrate that they care about the individual as a human being, not as a number or set of demographic checkboxes. No clients knew their providers before receiving H.O.P.E. services, and 8/8 clients indicated the importance of rapport building during screening. Women looked for signals that their provider authentically cared rather than merely going through the motions. These signals included nonverbal cues, tailored services, and follow-up. Notably, a lack of follow-up felt personal and made clients question the integrity of prior services. This further damaged strained client-healthcare system relationships.
Overlooked and adding up	4/8 women were pregnant in the sample, all of whom reported that pregnancy compounded their prior mental health concerns. 2/4 women reported how pregnancy led to a change in disease management, and 2/4 reported how societal glorification of pregnancy created an additional need for safe spaces to discuss the negative effects of pregnancy without judgment.

## Data Availability

The data that support the findings of this study were collected with the permission of the Mecklenburg County Public Health Department. Thus, restrictions apply to data sharing. In addition, due to ethical considerations for participants, data are not publicly available. The data are, however, available from the authors upon reasonable request and with the permission of Mecklenburg County Public Health Department.

## Abbreviations

The following abbreviations are used in this manuscript:

IC	Integrated behavioral health care
LIEM	Low-income and economically marginalized
CPOPCC	Client’s Perception of Provider’s Cultural Competence Instrument

## Appendix A

### Survey Questions

1. Which of the following services did you receive? (Circle all that apply).

My provider asked me questions about how I’ve been feeling recently, such as have I been feeling down, stressed, unhappy, hopeless, or depressed
Based on my responses, my provider recommended that I talk to the social worker/behavioral health provider on staff
I met with the social worker/Behavioral Health in-person or on the phone to talk about how I’ve been feeling
None of the above

2. Based on the services you circled above, how satisfied or dissatisfied are you with these specific services?

Strongly dissatisfied
Dissatisfied
Neither dissatisfied or satisfied
Satisfied
Strongly satisfied

3. How satisfied or dissatisfied were you with the overall services you received while at the clinic.

Strongly dissatisfied
Dissatisfied
Neither dissatisfied or satisfied
Satisfied
Strongly satisfied

4. What range reflects your current age?

18–24
25–34
35–44
45–54
55+

5. Do you identify as Hispanic/Latina?

Yes
No

6. Which of the following best describes your race? (Circle all that apply).

Asian
Black/African American
Native Hawaiian
Native Indian
White
Other (please specify) ____________________
Prefer not to answer

7. At which of the following clinics did you receive services? (Circle all that apply).

Clinic A
Clinic B
Clinic C
Clinic D
Clinic E

You indicated that while at the WIC of Family Planning clinic, you received at least one of the following services:

- Your provider asked you questions about how you’ve been feeling recently, such as have you been feeling down, stressed, unhappy, hopeless, or depressed?
- Based on your responses, your provider recommended that you talk to the social worker/behavioral health provider on staff
- You met with the social worker/Behavioral Health in-person or on the phone to talk about how you’ve been feeling

When answering the following questions, please refer ONLY to the times that you received the above services.
When the questions refer to your provider(s), consider ONLY the providers that delivered the specific services listed above (such as a nurse, physician’s assistant, nutritionist).
In the past 6 months, when receiving these services:

1. I was able to practice important routines and traditions related to my health.

Yes
No

2. I was comfortable sharing private and personal information with my provider(s).

Yes
No

3. I was understood and comforted after speaking with my care provider(s).

Yes
No

4. I had enough time to talk to my provider(s).

Yes
No

5. I was treated with respect.

Yes
No

6. I was consulted regarding my care.

Yes
No

7. I received care that fit my beliefs, work, and family.

Yes
No

8. The provider(s) worked with me to identify my needs and goals.

Yes
No

9. I am confident in my ability to get care for myself when needed.

Yes
No

10. My family or friends have access to resources to help me.

Yes
No

11. My provider(s) understood my needs.

Yes
No

12. The provider(s) was directly involved in my care.

Yes
No

13. I was connected with resources (ex: health information, support, services, etc.) available in my community.

Yes
No

14. Other people I consider important participated in my care.

Yes
No

## Appendix B

### Interview Guide

Screening Questions

You received services at either a WIC or Family Planning Clinic. While there, your provider asked you questions about how you’ve been feeling recently. They may have asked you questions like the following, “have you been feeling down or stressed? How have you been feeling about yourself? Have you felt like doing things you like? Have you had energy? Is there anything that bothers you or is making you unhappy or hopeless?” These questions all relate to your holistic wellness.

1. Can you tell me about this experience? How did it make you feel to be asked mental health questions while receiving services?
2. How comfortable did you feel being asked this question by your provider?
  a. Did you feel comfortable enough to answer honestly?

Referral Questions

Based on your responses to how you’ve been feeling, your provider (either a nutritionist, nurse practitioner, or PA) may have recommended that you talk to the social worker/behavioral health provider on staff. How did the conversation go with your provider?

1. Did you get the sense that this staff member would be able to support your holistic needs (such as childcare, housing, food assistance)? Why or why not?
2. Did you have any expectations for the behavioral health provider?

Consultation Questions

1. In three words, how would you describe your experience with the social worker/behavioral health provider?
2. Can you tell me more about your visit with [insert behavioral health provider’s name]?
  a. What did you gain?
  b. What suggestions for improvement?
  c. Were you able to access the community resources that you were referred to?
    - (If yes) Did you feel like the resources were appropriate and useful for you and your life?
    - (If yes) How could they be made more effective?

Whole-Person Care Questions

1. How have the services and resources you discussed (e.g., talking to someone about your feelings, seeing a social worker/BHP, being referred to community resources) shaped your overall perception of the public health clinics?

Integrating Natural Supports Questions

Let’s shift gears a little bit and focus now on wellness and non-medical supports that you use to promote your wellness. Wellness is not just physical wellness, it includes your spirituality, healthy relationships with friends and family, and positive mental health. Really wellness is whatever holistically makes you feel like your best self. In the survey you reported practices, resources, and communities that promote your wellness, such as religious groups, family, exercise, food, dancing, etc. Think about those when answering this question:

1. What do you do to keep yourself well?
2. How would you recommend incorporating these practices, resources, and communities into your treatment plan?
3. How might your recommendations improve your overall healthcare experience?

Barriers to Care Questions

1. What is the biggest barrier to accessing mental health care in your community?
2. What can be done to overcome this barrier?

## Appendix C

### Member Check Guide

First, we presented our preliminary findings. We use the following questions as a guide. However, we remained flexible to ensure that organic reactions, reflections, and suggestions could be provided by the participants.

1. What are your initial reactions or thoughts to what we just shared?
2. Were there any codes or themes that strongly resonated with you? If so, why?
3. Were there any codes or themes that did not fit your experience well? If so, why?
4. Is there anything else that you’d like to see reflected in the results from your experience?
